# Low Urinary Iodine Concentration among Mothers and Children in Cambodia

**DOI:** 10.3390/nu8040172

**Published:** 2016-04-05

**Authors:** Arnaud Laillou, Prak Sophonneary, Khov Kuong, Rathavuth Hong, Samoeurn Un, Chhoun Chamnan, Etienne Poirot, Jacques Berger, Frank Wieringa

**Affiliations:** 1 United Nations Children’s Fund (UNICEF), Maternal, Newborn and Child Health and Nutrition section, no11 street 75, Phnom Penh 12202, Cambodia; sun@unicef.org (S.U.); epoirot@unicef.org (E.P.); 2 National Nutrition Program, Maternal and Child Health Center, No 31A, Rue de France (St. 47), Phnom Penh 12202, Cambodia; sophonprak@gmail.com; 3 Department of Fisheries Post-Harvest Technologies and Quality Control (DFPTQ), Fisheries Administration, Ministry of Agriculture Fisheries and Forestry, 186 Preah Norodom Boulevard, Phnom Penh 12000, Cambodia; kuong.kh@gmail.com (K.K.); chhounchamnan@gmail.com (C.C.); 4 ICF International, 530 Gaither Road, Suite 500, Rockville, MD 20850, USA; rathavuth.hong@icfi.com; 5 Institute of Research for Development (IRD), UMR Nutripass IRD-UM2-UM1, Montpellier 3400, France; jacques.berger@ird.fr (J.B.); franck.wieringa@ird.fr (F.W.)

**Keywords:** Iodine, national survey, Cambodia, Demographic Health Survey, 2014

## Abstract

A 2014 national assessment of salt iodization coverage in Cambodia found that 62% of samples were non-iodized, suggesting a significant decline in daily iodine intakes. The Cambodian Micronutrient Survey conducted in 2014 (CMNS-2014) permitted obtaining national data on urinary iodine concentrations (UIC) to assess iodine status and whether iodized salt use had an impact. Urine samples were collected from mothers (*n* = 736) and children (*n* = 950). The median UIC was 63 µg/L and 72 µg/L in mothers and children respectively. More than 60% of mothers and their children had a UIC < 100 µg/L, thereby indicating a serious public health problem. Iodine status was significantly lower among mothers and children living in rural areas, belonging to the poorest socioeconomic category, or living in a household not using iodized salt. The limited enforcement of the legislation for iodized salt has resulted in a major decrease in the prevalence of iodized salt, which in turn has compromised iodine status in Cambodia. It is essential for the government to enhance enforcement of the iodized salt legislation, and implement short term strategies, such as iodine supplementation, to prevent an increase of severe complications due to iodine deficiency in the Cambodian population.

## 1. Introduction

The Royal Government of Cambodia initiated the National Sub-Committee for Control of Iodine Deficiency Disorders in 1996 to fight the high prevalence of iodine deficiency in Cambodia and associated functional and developmental abnormalities [1,2]. As recently as 10 years ago, slightly more than one in four (28%) of households were using iodized salt in Cambodia [3]. Since the 2003 Government’s Sub-Decree No. 69 on mandatory iodization of salt and several related regulations, Cambodia’s supply of iodized salt has increased greatly to reach approximately 70% of households around 5 years ago [3]. Unfortunately, recent evidence suggests that the proportion of non-iodized salt collected from markets in Cambodia increased from only 22% in 2011 to 62% in 2014 [4]. This decrease coincided with the moment that international organizations ceased to support the procurement of iodine. Furthermore, the levels of iodine added to salt are too low, with 99.6% of the coarse salts and 82.4% of the fine salts tested having iodine levels outside the government standards of 30–60 ppm [4]. Before the introduction of the iodization program in Cambodia in the late 1990’s, almost 20% of primary school children were affected by goiters [5]. It is important to know whether the current low prevalence of iodized salt that is available at the community level affects urinary iodine concentration (UIC) in the Cambodian population. Countries can face various challenges related to iodine insufficiencies; once more than 50% of households do not have adequate access to iodized salt, the (re-)appearance of new cases of goiters and cretinism is likely to become increasingly common [6,7,8].

Recent evidence from school children in one province (Kampong Speu) in Cambodia suggests a significant decrease in UIC among schoolchildren [9]. According to a survey implemented by the French Institute IRD in 2014, the UIC median was 167 μg/L, while in 2011, a survey led by UNICEF reported a median UIC of more than 230 μg/L within the same province and population group [10]. In Cambodia, data on iodine concentration in urine are sparse or carried out on limited samples of populations. However, the 2014 Cambodian Demographic Health Survey (2014 CDHS) and the Cambodian National Micronutrient Survey 2014 (CNMS-2014) considered this issue, and in doing so, presented the opportunity to obtain national representative data on UIC levels in mothers and children. The present study was carried-out on a subset of mothers and children from the 2014 CDHS to provide information on UIC at the national level in relation to their living area (urban/rural), access to branded iodized salt, ownership of an equity card, and socio-economic status.

## 2. Experimental Section

### 2.1. Study Design and Sampling

The 2014 CDHS is a nationally representative sample of children, aged 0–5 years, and women and men between 15 and 49 years of age. A representative sample of 16,356 households was selected for the 2014 CDHS. The sample was selected in two stages. In the first stage, 611 villages (also known as clusters or enumeration areas) were selected with probability proportional to village size. Village size is defined as the number of households residing in the village. Then, a complete mapping and listing of all households existing in the selected villages was conducted. The resulting lists of households served as the sampling frame for the second stage of sample selection, with households randomly selected for participation in the survey.

To provide data on the current micronutrient status of the Cambodian population, a national micronutrient survey (CNMS-2014) was conducted in parallel with the 2014 CDHS. The CNMS-2014 revisited 1/6 of the main survey clusters of the 2014 CDHS and collected additional data on the micronutrient status of women and children under 5 years of age. In the selected households, urine samples were collected from women who had given birth in the five years preceding the survey and from their children aged 6–59 months. The sample size for this study was estimated to obtain a precision of 3% at the national level and 5% at urban and rural level. The study aimed to recruit a sample of 935 mothers and 1096 children, accounting for 20% non-compliance.

The women, who were between 15 and 49 years of age, were selected according to the following criteria: (i) mother of a child aged 6–59 months; (ii) from households having previously participated in the 2014 CDHS; (iii) no evidence of severe or chronic illness, and; (iv) having consented to participate in the micronutrient survey.

The criteria to participate in the survey for children less than five years of age were as follows: (i) from households having previously participated in the 2014 CDHS; (ii) between 6 and 59 months of age; (iii) no evidence of severe or chronic illness (such as high fever, on intra-venous drops, taking tuberculosis treatment), including congenital abnormalities, mental or severe physical handicap, and; (iv) written informed consent signed by at least one parent.

Socio-economic status was calculated using the Demographic Health Statistic (DHS) Wealth Index [11] to divide households surveyed into the standard three socio-economic groups: the “poorest” (category 1), the “middle” (category 2), and the “richer” (category 3). The Wealth Index was constructed from recorded data on household assets such as tables, chairs, refrigerators, air conditioners, and beds, and also from housing conditions (the materials used for the house’s floor, roof, and main walls) and facilities (energy for cooking, electricity and latrines), whilst income and expenditures were not used.

### 2.2. Urine Sampling and Analysis

Data were collected at each house in each cluster in the morning. After checking for household and individual codes to match those of the 2014 CDHS, a short questionnaire was taken by the mother/caretaker. Mothers and one of their children (under 5 years of age) were provided with 1 container each for the collection of urine. After obtaining the urine samples, they were stored in cool packs (blue ice), and transported in the afternoon to the provincial health center where they were kept frozen at −18 °C. They were then transported on ice packs within 4 days to the Department of Fisheries Post-harvest Technologies and Quality control (DFPTQ), Ministry of Agriculture, Forestry and Fisheries, Phnom Penh. The samples were thereafter frozen at −20 °C.

The urine samples were transported on dry ice to the Institute of Nutrition at Mahidol University, where they were stored at −20 °C while waiting to be analyzed. At Mahidol, the iodine concentration in urine was measured using the Pino modification of the Sandell-Kolthoff reaction [12]. External control was provided by the Laboratory of Human Nutrition at the Swiss Federal Institute of Technology Zurich using the same method, and the inter-laboratory coefficient of variation at a mean UI of 100 μg/L and 212 μg/L (*n* = 10 each) was less than 8%.

### 2.3. Salt Analysis

A small amount of salt (half a tea spoon) was taken from each household visited and tested for iodine presence with a salt rapid test kit (RTK). This qualitative iodine estimation in salt is based upon the reaction between starch and iodine to form starch-iodine complex. This test solution contains an acidic buffer and a reducing agent, which convert potassium iodate (KIO3) to elemental iodine (I2). This elemental iodine reacts with the amylose of the starch to result in a visible blue-black color [13].

### 2.4. Statistical Analysis

Data analysis was performed using SPSS software (SPSS, V20; IBM, Chicago, Illinois, USA). In addition to quantitative analysis of the results, values were also categorized into: (I) deficiency as indicated by low UIC (UIC < 100 µg/L); (II) normal UIC (UIC = 100–299.9 µg/L); and (III) excessive UIC (UIC ≥ 300 µg/L) [14]. UIC was not normally distributed (Kolmogorov-Smirnov test) and therefore only non-parametric tests were performed. Median and interquartile range values of the iodine concentrations are presented and disaggregated by wealth index, area of living, access to branded iodized salt, access to iodized salt (according to rapid test kits used during the 2014 CDHS), access to equity card (Households identified as poor in Cambodia receive an “Equity Cards”. Poor households can use the equity cards to access a range of services provided by the Government and other organizations. For example, in the health sector, poor households can use the equity card to receive free health care. Other services and benefits include education scholarships for girls and boys from poor households, access to public works programs that provide cash or food, and food aid, *etc*.), and age groups for children. Iodine concentrations (continuous variables) were compared by groups by using the non-parametric Mann-Whitney U-test (two groups) or Kruskal-Wallis (k groups) for independent variables. The odds ratio was analyzed to examine the association between having a low UIC (below 100 µg/L) and several independent factors. Multivariate logistic regression analyses for risk factors associated with UIC < 100 µg/L were carried out. Variables in the model were selected through a backward stepwise conditional approach. Variables not significant in the model (*p* > 0.05) were excluded. Group age for children was kept in the model even if not significant. The covariates used to build the model were: age for children only (<24 months, above or equal to 24 months), salt test at household (positive for iodine and not positive for iodine), access to equity card (yes and no), and packaging of salt at household (labelled as iodized and no labelling), living area (urban/rural), and wealth quintile (poor and rich). Results were considered significant at *p* < 0.05.

### 2.5. Ethical Issues

The Scientific Committees of the Ministry of Health (Phnom Penh, Cambodia) reviewed and approved the study protocol. All households and women were informed verbally and in writing about the aims and procedures of the study, and informed consent was obtained from all women and children (via their mother or guardian) before enrollment.

## 3. Results

In total, 736 women and 950 children consented to the study and provided urine samples, with 21% living in urban areas, and 79% living in rural areas. Among mothers surveyed, 42.6% were classified in the “poorest” category (category 1), 17.8% in “middle” (category 2), and 39.4% in the “richest” (category 3). Within our sub-sample from the DHS, 75.6% of the salt tested with an iodine spot test indicated the presence of iodine.

Median UIC was significantly higher among mothers according to their socioeconomic categories (*p* < 0.001), with a median UIC of 55 µg/L for the poorest category (25th–75th percentile: 27 µg/L–90 µg/L), and 75 µg/L for the richest category (25th–75th percentile: 43 µg/L–121 µg/L). Significant differences were observed in median UIC according to place of living and access to iodized salt (Table 1). Bivariate analyses showed that iodine status of women was significantly associated with socioeconomic status, urban or rural location, and access to iodized salt.

Using the WHO cut-offs, 74.6% of the mothers were considered to have a low UIC (below 100 µg/L, Table 2), while 2% of the mother had a high UIC respectively. The prevalence of low UIC (less than 100 µg/L) was significantly higher for those who were living in a rural area, belonging to the poorest and middle social-economic class, or using salt in a household that tested negative for iodine (Table 2).

Similar trends were observed amongst children (Table 3). Significant differences were observed in median UIC according to location, age groups, access to iodized salt, and access to the Cambodian equity card. Bivariate analyses showed that median UIC was significantly lower in children living in rural areas, belonging to the poorest socioeconomic category as compared to the richest category, or for those living in a household using salt that tested negative for iodine (Table 3).

Results indicated that 63.7% of children had UIC below 100 µg/L. The prevalence of UIC below 100 µg/L increased significantly with age, starting at 53.3% for children less than a year of age and increasing to 67.9% for the oldest categories (Table 4). Children from the highest socioeconomic group (group 3) had the lowest prevalence of UIC below 100 µg/L (48.2%) and the highest prevalence of UIC above 300 µg/L (6.0%).

The unadjusted odds of having a UIC below 100 µg/L were almost halved for mothers and reduced by two thirds for children if they belonged to the richest socio-economic category as compared to the poorest (respectively Odd Ratio (OR): 0.55 (95% CI: 0.38–0.80) and 0.33 (95% CI: 0.24–0.44)). The unadjusted odds of having a UIC below 100 µg/L was 2.6 times higher among rural children compared to urban children (OR: 2.64 (95% CI: 1.92–3.63)), and 2.3 times higher among rural mothers compared to urban mothers (OR: 2.29 (95% CI: 1.56–3.35)). Similar figures were observed if the children or mothers had no access to iodized salt, as the unadjusted odds of having a UIC below 100 µg/L were doubled (OR: 2.06 (95% CI: 1.47–2.89)) for children (OR: 2.06 (95% CI: 1.47–2.89)) and mothers (OR: 2.21 (95% CI: 1.42–3.45)) without access in comparison to the ones with access. The odds among children and mothers from middle socioeconomic group of having a UIC below 100 µg/L was respectively 1.66 (CI: 1.03–2.69) and 2.76 (CI: 1.87–4.07) higher than if they belonged to the richest category.

The multivariate model analysis showed that only access to equity card for children was not significantly influencing the odds of having a UIC below 100 µg/L while living in a rural area, having no access to iodized salt, and being from the poorest socioeconomic groups; being older than 24 months significantly increased the odds of having a UIC below 100 µg/L by more than 1.45 (Table 5). Among mothers, only living in rural area and having no access to iodized salt significantly increased the odds.

## 4. Discussion

To our knowledge, this study is the first in over a decade to report national representative data on iodine status in Cambodia. Our results are worrisome as the median UIC is below 100 µg/L for both mothers and children. More than 60% of mothers and their children had UIC below 100 µg/L, which is indicative of iodine deficiency. This is an urgent public health problem, as this condition is often associated with serious health and developmental consequences. Considering the high prevalence of UIC below 100 µg/L, it is likely to also be present in women entering pregnancy and thereby presents subsequent risks to impaired fetal development [15].

The current study results are consistent with previous study findings that demonstrate the prevalence of adequately iodized salt use is low in Cambodia [4]. Since 2001, WHO/UNICEF/ICCIDD showed that median UIC is highly sensitive to any changes in the iodized salt programs when this condiment is the main daily source of iodine intake [16]. In 2014, UNICEF and its partners showed that more than 60% of the salts checked did not meet the national standard [4].

The high prevalence of low UIC in children, which increases even further with age, is also a major concern. It is recognized that 39% of children under 5 years in low-income and middle-income countries do not reach their mental potential with iodine deficiency as one of the contributing reasons [17,18]. During the first two years of life, breastfeeding has been recognized as being essential for many reasons, including the reduction of chronic diseases among young children. We should not underestimate the contribution of breastmilk to the iodine intake of young children [19]. Breastmilk could be supporting the prevention of inadequate UIC observed among more than 50% of the children aged 6–23.9 months if their mothers are consuming adequately iodized salt. It is clear that in Cambodia the decline of breastfeeding after 6 months (from 85.8% among 6.0 to 11.9 months to 57.6% from 12.0 to 23.9 months) [20] coupled with unsatisfactory compliance of iodized salt [4] could explain a part of the 18 µg/L decrease of the median UIC in older children (12–23.9 months). But breastfeeding and iodized salt consumption alone will not provide enough iodine to meet a child’s needs from the age of 6 months especially if the mother is marginally iodine sufficient, unless complementary foods are fortified with iodine as well [21]. WHO recommends that children from birth to 24 months of age should receive iodine through breast milk from a mother supplemented with iodine (salt or iodine supplements) and, in elder children, through complementary foods fortified with iodine [21].

With increasing age, children depend more and more on family foods for their iodine intake and therefore have other potential source of iodine (including fish sauce and several seasonings that use iodized salt during the production process). Our study shows that the median UIC continues to decrease for older children to reach a median UIC of only 55 µg/L for children older than 5 years old. The limited availability of food varieties that naturally contain iodine in Cambodia and the lack of adequately iodized salt or any other processed foods (such as condiments) are key factors for low iodine intake of older children, and consequently impact UIC. The presence of iodine in salt in the household was related to a higher median UIC among children and women compared to households without access to iodized salt, showing the potential impact of food or condiments fortified with iodine.

The study also notes that 48% of children from the richest socioeconomic group have UIC below 100 µg/L (compared to 74% among the poorest groups). Targeting services or demands to the poor, as it is done for health (health equity funds [22]) and other sectors will not be sufficient. Ideally, equity suggests that the entire Cambodian population should not only have the same access to services, but also to essential nutrition interventions, such as iodized salt, to attain their full health and development potential. Unfortunately, our study shows that the median UIC from children living in the poorest families is less than half of what is observed in the richest quintile (51 µg/L *versus* 104 µg/L). Even children from middle quintile households have a 2.8 higher odds ratio of having a median UIC below 100 µg/L in comparison to the richest. Thus, ensuring equity in iodine nutrition is critical. Unpackaged and unlabeled coarse and refined salt are often available at markets and sold at a cheaper price that targets those most in need of proper iodized salt. It is essential to sustain strong enforcement from the government on the approved legislation and ensure that everyone has access to affordable and adequately iodized salt.

Even if the prevalence of children and mothers having UIC above 300 µg/L are low (less than 5%), it is essential to avoid any increase of this prevalence (which is considered to be excessive) [13]. In 2008 and 2011, the prevalence of school aged children having UIC above 300 µg/L were 32% and 22%, respectively [23]. Therefore, while the government needs to ensure that salt is well iodized, it is important to review the standards to be in line with the recent WHO recommendation [24] which will significantly decrease the actual standard from 30 to 60 ppm to 14 to 22 ppm. This reduction will help to minimize the population with UIC above 300 µg/L, thereby helping to reduce the number of individuals at a higher risk of adverse related health consequences (including iodine-induced hyperthyroidism and autoimmune thyroid diseases).

To enforce policy on salt iodization, the government needs to use the best tools available. According to the rapid test kits (RTK) implemented during DHS, and often used in Cambodia, three out of four of the samples tested positive to iodine. A study in Nepal confirmed the accuracy of rapid test kits as a qualitative tool [25]; other tools are available to quantitatively determine salt iodine levels [26]. We could therefore extrapolate that the DHS was detecting a large amount of salt with levels of iodine that were insufficient to have a significant impact on UIC. Alternatives such as the WYD Checker or the iCheck Iodine, both giving a quantitative measurement of iodine in the salt, should be investigated for on-going monitoring in Cambodia [26]. Finally, due to the current global guidance to reduce salt intake from 15 g/day per capita in Cambodia [27] to <5 g/day to prevent heart disease and stroke [28], it is important to ensure that salt is properly iodized or Cambodians will further reduce their iodine intake thereby increasing the risk of any related disorders.

*Limitations*: UIC is associated with large intra-individual variation, making it only suitable for use in groups. Furthermore, the WHO cut-offs for UIC have been validated in school aged children (6 years or older) and pregnant women [14]. Therefore, we have decided to extrapolate the cut-offs of school aged children for our different groups. Our recommendation should be strengthened with further research as required UIC for WRA may be below 100 µg/L [29]. No conclusion can be made at the individual level as ten repeat collections for urinary iodine from spot samples or 24-h samples are needed to reliably estimate individual iodine status in women and children [30]. As recently published, additional data should be collected to determine the best manners in which to assess iodine intake both on an individual basis and across different populations, including those most vulnerable to iodine deficiency (namely pregnant and lactating women, and children) [31].

## 5. Conclusions

The low median UIC (below 100 µg/L) in 2014 among mothers and children is calling for urgent actions. In a recent paper [4], one of the bottlenecks of the Cambodian IDD control program was the burden (additional cost) of the actual salt iodization standard in Cambodia (30–60 ppm) compared to the neighboring salt producing countries where different legislations are in place. With an estimated daily consumption of 15 g of salt per day in Cambodia [4], the government could lower their standard to be in line with new WHO recommendations [23]: iodine levels of 14–20 ppm at production level assuming consumption 14–10 g/salt/day. This new standard could have a positive impact on the compliance of producers and consequently on UIC. It would also contribute to limit any potential increase of the percentage of population with UIC above 300 µg/L, as it was observed to be a potential cause for concern for segments of the population in 2011 (22% of the pre-school children [23]). The current poor iodine status is due to non-compliance with national standards, rather than due to insufficient iodine levels mandated in salt. Reducing iodine levels would also make iodization cheaper for salt producers/importers and could be presented as a measure “supporting the salt industry”. In addition, short term strategies to prevent the increase of severe complications due to low UIC should be explored, including iodine supplementation for at risk population until salt is well iodized (see recommendation from previous paper [4]), as well as ensuring that adequate monitoring system are in place.

## Figures and Tables

**Table 1:** Bivariate relationships between median urinary iodine concentrations (UIC) and living area, wealth quintiles, access to iodized salt, and access to equity card among mothers.

	Urinary Iodine Concentration			
	Median (µg/L)	25th–75th Percentile (µg/L)	*N*	*p* ^1^
**Total**	63	(33; 101)	736	-
**Living area**				
Urban	78	(45; 130)	151	*<0.001*
Rural	58	(31; 93)	585	
**Wealth quintiles**				
Poorest	55	(27; 90)	315	*<0.001*
Middle	56	(31; 90)	131	
Richest	75	(43; 121)	290	
**Access to iodized salt**				
Salt tested positive	68	(36; 110)	554	*<0.001*
Salt tested negative	49	(21; 77)	176	
**Packaging**				
Package labelled as iodized	76	(43; 119)	250	*<0.001*
No labelling	53	(26; 89)	334	
**Access to equity Card**				
Equity card seen	55	(30; 93)	142	*<0.001*
Equity card but not seen ^2^	73	(39; 106)	24	
No equity card	65	(32; 102)	570	

Note: ^1^ non parametric tests were performed as Kolmogorov-Smirnov test showed that the distribution is not normal. Therefore Mann-Whitney has been performed for 2 independent samples and Kruskal-wallis for k-independent samples; ^2^ the household claimed to have an equity card but didn’t show it the interviewer.

**Table 2:** Bivariate relationships between the prevalence of iodine level in urine and living area, wealth quintile, access to iodized salt, and access to equity card among mothers.

%	Prevalence of Iodine Level in Urine					
	Less 50 µg/L	50–99 µg/L	100–299 µg/L	≥300 µg/L	*N*	*p* ^1^
**Total**	40.2	34.4	23.9	1.5	736	-
**Living area**						
Urban	28.5	32.5	37.7	1.3	151	*<0.001*
Rural	43.1	34.9	20.3	1.7	585	
**Wealth quintiles**						
Poorest	44.8	34.6	17.8	2.8	315	*<0.001*
Middle	48.1	29.8	21.4	0.7	131	
Richest	31.7	36.2	31.7	0.4	290	
**Access to iodized salt**						
Salt tested positive	37.0	34.3	27.1	1.6	554	*<0.001*
Salt tested negative	51.1	34.1	13.6	1.2	176	
**Packaging**						
Package labelled as iodized	32.0	33.6	33.2	1.2	250	*<0.001*
No labelling	47.9	32	18.3	1.8	334	
**Access to equity Card**						
Equity card	42.8	32.5	22.3	2.4	166	*0.573*
No equity card	39.5	34.9	24.4	1.2	570	

Note: ^1^ non parametric tests were performed as Kolmogorov-Smirnov test showed that the distribution is not normal. Therefore Mann-Whitney has been performed for 2 independent samples and Kruskal-wallis for k-independent samples.

**Table 3:** Bivariate relationships between median UIC and living area, wealth quintiles, age groups, access to iodized salt, and access to equity card among children under 5 years of age.

	Urinary Iodine Concentration			
	Median (µg/L)	25th–75th Percentile (µg/L)	*N*	*p* ^1^
**Total**	72	(36; 136)	950	-
**Living area**				
Urban	112	(52; 172)	201	*<0.001*
Rural	64	(33; 122)	749	
**Wealth quintiles**				
Poorest	51	(27; 106)	410	*<0.001*
Middle	61	(30; 110)	175	
Richest	104	(53; 167)	365	
**Age groups**				
6–11 months	90	(50; 179)	90	*0.002*
12–23 months	72	(40; 139)	156	
24–59 months	72	(34; 129)	565	
60+ months	55	(34; 126)	137	
**Access to iodized salt**				
Salt tested positive	75	(39; 141)	725	*<0.001*
Salt tested negative	62	(30; 101)	218	
**Packaging**				
Package labelled as iodized	92	(46; 162)	309	*<0.001*
No labelling	60	(29; 117)	431	
**Access to equity Card**				
Equity card seen	58	(27; 116)	197	0.007
Equity card but not seen ^2^	71	(37; 138)	35	
No equity card	77	(38; 140)	718	

Note: ^1^ non parametric tests were performed as Kolmogorov-Smirnov test showed that the distribution is not normal. Therefore Mann-Whitney has been performed for 2 independent samples and Kruskal-wallis for k-independent samples; ^2^ the household claimed to have an equity card but didn’t show it the interviewer.

**Table 4:** Bivariate relationships between the prevalence of iodine level in urine and living area, wealth quintile, access to iodized salt, age groups, and access to equity card among children under 5 years of age.

%	Prevalence of Iodine Level in Urine					
	Less 50 µg/L	50–99 µg/L	100–299 µg/L	≥300 µg/L	*N*	*p* ^1^
**Total**	36.4	27.3	31.7	4.6	950	-
**Living area**						
Urban	20.4	24.9	46.8	7.9	201	*<0.001*
Rural	40.7	27.9	27.6	3.8	749	
**Wealth quintiles**						
Poorest	48.3	25.6	22.0	4.1	410	*<0.001*
Middle	38.9	33.1	25.1	2.9	175	
Richest	21.9	26.3	45.8	6	365	
**Age groups**						
6–11 months	24.4	28.9	35.6	11.1	90	*0.005*
12–23 months	32.1	31.4	32.1	4.4	156	
24–59 months	37.2	27.4	31.3	4.1	565	
60+ months	46.7	21.2	29.1	3.0	137	
**Access to iodized salt**						
Salt tested positive	34.5	25.5	35.0	5.0	725	*<0.001*
Salt tested negative	41.3	33.5	21.6	3.6	218	
**Packaging**						
Package labelled as iodized	27.2	26.2	40.1	6.5	309	*<0.001*
No labelling	44.1	27.8	26.2	1.9	431	
**Access to equity Card**						
Equity card	43.5	28.4	24.1	4.0	232	*0.002*
No equity card	34.1	26.9	34.1	4.9	718	

Note: ^1^ non parametric tests were performed as Kolmogorov-Smirnov test showed that the distribution is not normal. Therefore Mann-Whitney has been performed for 2 independent samples and Kruskal-wallis for k-independent samples.

**Table 5:** Multivariate model analysis: binary logistic regression analysis for the risk factors associated with UIC below 100 µg/L (Variables in the model were selected through a backward stepwise conditional approach. Variables not significant in the model (*p* > 0.05) were excluded. The covariates used to build the model were: age for children only (<24 months, above or equal to 24 months); salt test at household (positive for iodine and not positive for iodine), access to equity card (yes and no), and packaging of salt at household (labelled as iodized and no labelling), living area (urban/rural), and wealth quintile (poor and rich). Results were considered significant at *p* < 0.05).

	Degrees of Freedom (DF)	*p* Value	Adjusted Odds	95% Confident of Interval (CI) for Odds
**Children**				
Rural area	1	**0.026**	**1.56**	**1.06–2.32**
Richest category	1	**0.001**	**0.44**	**0.30–0.64**
≥24 months of age	1	**0.037**	**1.45**	**1.02–2.06**
Salt tested negative for iodine	1	**0.021**	**1.61**	**1.07–2.41**
Packaging not labelled as iodized	1	**0.033**	**1.45**	**1.03–2.03**
Access to equity card	1	0.739	1.07	0.72–1.60
**Women**				
Rural area	1	**0.043**	**1.62**	**1.02–2.59**
Richest category	1	0.195	0.74	0.47–1.17
Salt tested negative for iodine	1	**0.048**	**1.67**	**1.00–2.78**
Packaging not labelled as iodized	1	0.103	1.41	0.93–2.12
Access to equity card	1	0.357	1.26	0.77–2.04
